# How the negative role of authoritarian leadership leads to quiet quitting: the moderated mediating role of involuntary presenteeism

**DOI:** 10.3389/fpsyg.2026.1821724

**Published:** 2026-07-03

**Authors:** Bowen Shan, Xiu Jin

**Affiliations:** Department of Business Administration, Gachon University, Seongnam, Republic of Korea

**Keywords:** authoritarian leadership, involuntary presenteeism, job burnout, moderated mediating model, quiet quitting

## Abstract

Quiet quitting emerged as a widespread form of employee withdrawal in the post-pandemic era, reflecting shifting work norms, unmet psychological needs, and deteriorating relationships. Although previous research has highlighted its detrimental consequences for organizational functioning, empirical evidence on its antecedents and underlying mechanisms remains limited. Drawing on the conservation of resources and self-determination theories, this study investigates how authoritarian leadership leads to quiet quitting among employees of Chinese small- and medium-sized enterprises (SMEs). Specifically, it proposes and tests a moderated mediation model in which authoritarian leadership increases quiet quitting through its positive effect on job burnout, whereas involuntary presenteeism strengthens the effect of burnout on quiet quitting. Focusing on Chinese SMEs, this study provides contextually grounded evidence on how employees interpret and respond to authoritarian leadership. To achieve this research purpose, data were collected from 363 employees working in Chinese SMEs. The results demonstrate that job burnout serves as a key psychological mechanism linking authoritarian leadership to quiet quitting. Furthermore, involuntary presenteeism amplifies the transition from job burnout to quiet quitting by exerting a positive reinforcing effect on this pathway and thereby intensifying the overall negative process. By uncovering these mechanisms, this study contributes to a deeper understanding of the harmful consequences associated with authoritarian leadership and clarifies the emergence and evolution of quiet quitting in the Chinese cultural context.

## 1 Introduction

Against the backdrop of changing work norms intensified by the COVID−19 pandemic, quiet quitting gained prominence in both academic and public discourse as a response to perceived shifts in psychological contracts (Williams and Kayaoglu, 2020). Unlike formal resignation, quiet quitting employees choose to meet only the minimum job requirements while psychologically disengaging themselves from their roles (Scheyett, 2023; Karrani et al., 2024). Due to experiencing greater disruptions in their careers, such as layoffs, inflation, and recession, compared to previous generations, many Millennials and Gen Z individuals have become disillusioned with their career prospects, prompting them to rethink their relationships with work, become increasingly disengaged, and ultimately consider quitting their jobs (Kruse and MDiv, 2023). Although quiet quitting may be beneficial for the individual, it is detrimental for organizational performance, particularly in terms of reduced morale, productivity, engagement, and overall success (Dillard et al., 2025). Identifying quiet quitting is inherently difficult because employees often conceal such actions, making the phenomenon likely to be considerably more prevalent than documented (Gün et al., 2025a). Recent evidence further suggests that quiet quitting may become increasingly common among organizations years ahead (Galanis et al., 2024a,b). Bennett et al. (2025) show, when employees exhibit quiet quitting, coworkers often interpret minimal engagement as an indication that the individual prioritizes personal interests and disregards the implicit norms of group reciprocity. When coworkers perceive such withdrawal, they tend to reduce their supportive behaviors and display higher levels of workplace incivility. Therefore, understanding the factors that give rise to quiet quitting and developing effective strategies to curb such behavior are essential for organizational development.

Quiet quitting initially gained considerable attention due to its viral spread on social media and is regarded as the second wave of workplace transformation following the “Great Resignation” (Harris, 2025). More than half the employees in the United States can be considered quiet quitters, according to one recent study (Wu and Wei, 2024). Meanwhile, other scholars note that this phenomenon gained attention in several Asian countries, emphasizing that culturally specific expressions—such as “tang ping” in China—already exist to describe similar behaviors (Campton et al., 2023). Hamouche et al. (2023) suggest that quiet quitting is not directly caused by the COVID-19 pandemic but rather has been accelerated and amplified by it. As the authors note, although quiet quitting has been described in terms such as reduced work engagement and strict adherence to formal job requirements, the phenomenon itself appears to have existed long before the pandemic. However, its prevalence increased significantly in the post COVID-19 period, leading commentators to attribute the observed increase to the pandemic. Amid the growing academic interest, scholars across disciplines have increasingly acknowledged the significance of quiet quitting, frequently highlighting its rising prevalence and diverse impacts on organizational functioning (Harris, 2025). Although various definitions exist in the literature, employees who engage in quiet quitting are typically characterized as employees who hold a clear intention to leave their job yet remain in their positions because of particular situational constraints, and consequently perform only the minimum required aspects of their job duties (Gün et al., 2025a). This study explores quiet quitting among employees in the Chinese economic context. Focusing on small and medium-sized enterprises (SMEs) in China, this study investigates the antecedents and underlying mechanisms that give rise to quiet quitting among employees in this unique organizational and cultural setting.

Rooted primarily in hierarchical and high-power-distance cultural contexts, authoritarian leadership is characterized by a strong emphasis on control and dominance coupled with restricted autonomy for subordinates (Cheng et al., 2004; Harms et al., 2017). Although authoritarian leadership may facilitate short-term compliance, it has been linked to increased stress, diminished intrinsic motivation, and greater burnout (Zhu et al., 2023). Authoritarian leaders reinforce their personal dominance over subordinates by exploiting power imbalances, centralizing authority, and maintaining control through unilateral decision making (Tsui et al., 2004). Dominance-focused authoritarian leadership has been shown to diminish project team members’ psychological empowerment, evoke negative emotions such as fear and uncertainty, suppress proactive motivation and self-initiative, and ultimately impede both task and innovation performance (Liu et al., 2024). This leadership style is linked to eroded trust (Parlar et al., 2022), increased silence behavior (Zhu et al., 2025), and heightened resistance to change (Mohamed et al., 2025). This study suggests that authoritarian leadership may not only independently predict quiet quitting but also exacerbate the harmful effects of hindrance stressors on employee burnout and quiet quitting.

Furthermore, job burnout is a pivotal mediating mechanism linking adverse workplace conditions to quiet quitting (Gün et al., 2025a). Burnout, especially emotional exhaustion, is a state of chronic psychological depletion that frequently precedes employees’ withdrawal from non-mandatory tasks (Maslach et al., 2001). Employees who experience job burnout often enter a vicious cycle where various factors interact and amplify one another, resulting in ongoing psychological distress and deteriorating job performance (Galanis et al., 2024a,b). A growing body of research demonstrates that job burnout negatively impacts employees’ work lives, contributing to depressive symptoms (Huo et al., 2021) and reduced job satisfaction (Alzailai et al., 2021). Moreover, burnout is one of the most significant predictors of employees’ intentions to leave their jobs (Özkan, 2022). Despite growing interest in burnout’s consequences, its mediating role in predicting quiet quitting and how involuntary presenteeism could mediate this pathway, remain untested.

While prior research has identified antecedents, such as role ambiguity (Wu and Wei, 2024) and organizational justice (Georgiadou et al., 2025), relatively little is known about the underlying psychological mechanisms and contextual pressures through which these or other stressors contribute to quiet quitting. Job burnout, characterized by emotional exhaustion, cynicism, and reduced professional efficacy, is a key predictor of employee withdrawal from non-mandatory work activities (Ju and Pak, 2024). However, the intensity of this relationship may fluctuate based on contextual factors that further limit employees’ capacity to safeguard or replenish their resources. Involuntary presenteeism refers to the intention to attend work while ill due to external pressures or demands (Van Waeyenberg, 2024). According to Deci and Ryan’s (2000) self-determination theory, this behavior is driven by controlled motivation, in which employees feel compelled to comply with external demands rather than act out of their own volition. Because involuntary presenteeism reflects situations in which employees are unable to withdraw from work to recuperate, it may intensify the effect of burnout on tendencies toward quiet quitting. In these circumstances, employees who are already resource-depleted may increasingly adopt minimal-effort strategies to conserve what remains, indicating that involuntary presenteeism serves as a crucial boundary condition in the relationship between burnout and quiet quitting.

Building on these research gaps, this study has four primary objectives. First, quiet quitting grew into a prominent research topic in recent years and numerous studies demonstrate its detrimental consequences for organizations. However, researchers have insufficiently examined the antecedents and underlying mechanisms that give rise to quiet quitting. When employees perceive that management does not provide adequate support, their work-related stress increases, which in turn leads to psychological disengagement from the organization. Kim and Sohn (2024) describe this form of disengagement as manifesting in quiet quitting. Mahand and Caldwell (2023) note that although a variety of organizational and individual factors shape quiet quitting, a lack of employee autonomy and career development opportunities, a diminished sense of personal value, and a decline in organizational trust ultimately cause it most commonly. When employees perceive that the organization places less importance on their contributions, or when they experience restricted opportunities for career growth and insufficient recognition, they may gradually develop a sense of emotional disengagement. Organizational trust deterioration further reinforces this disengagement. Employees begin to believe that the organization no longer acts in its best interests and fails to uphold the expected norms of reciprocity. Employees, therefore, do not quit because of laziness or a lack of work ethics. Rather, unmet psychological needs and the progressive breakdown of workplace relationships reflect it (Mahand and Caldwell, 2023). This study employs empirical analysis to validate how authoritarian leadership positively affects quiet quitting, and it draws on conservation of resources theory to clarify the theoretical pathway through which this influence occurs.

Second, this study investigates the mediating role of job burnout in the relationship between authoritarian leadership and quiet quitting. Prior research shows that employees who experience resource depletion and develop job burnout often adopt quiet quitting as a psychological defense mechanism, limit their work engagement to the minimum required level, and refrain from any additional effort (Jin et al., 2025). However, the specific psychological process through which employees exposed to authoritarian leadership develop job burnout and subsequently quit is not yet fully established. This study proposes that harmful leaders undermine employee well-being through exposure. An individual experiences emotional exhaustion, a negative psychological condition that arises when their emotional resources are nearly depleted and typically manifests as fatigue, anxiety, depressed mood, and a sense of diminished enthusiasm. Authoritarian leaders act as significant stressors that intensify emotional exhaustion. This not only lowers employees’ performance and satisfaction, impairing their work states, but also causes spillover effects in their personal lives and ultimately triggers quiet quitting (Peng and Huang, 2024).

Third, this study examines how involuntary presenteeism moderates the relationship between job burnout and quiet quitting. Quiet quitting gained substantial attention during the Covid-19 period, and involuntary presenteeism captured the phenomenon in which employees felt compelled by external pressures to attend work, even when unwell. Recent research has emphasized the importance of integrative models that simultaneously consider mediating and moderating mechanisms in explaining employee behavior. For instance, Rubel et al. (2025) demonstrate that leadership influences employee ethical voice behavior through psychological mechanisms such as person–job fit and person–organization fit, while being contingent upon contextual factors such as co-workers’ ethical support. Building on this perspective, the present study develops a moderated mediation model to examine how authoritarian leadership affects quiet quitting through job burnout, with involuntary presenteeism serving as a key boundary condition. Changes in work organization following Covid-19, together with the traditional ways in which work has been structured within organizations, may contribute to the continued rise in quiet quitting in the coming years (Hamouche et al., 2023). This study argues that high levels of involuntary presenteeism experienced by employees strengthen the positive effect of job burnout on quiet quitting through an enhancing moderating effect.

Fourth, this study focuses on the employees of Chinese SMEs. In high-power-distance contexts (Rui et al., 2024), authoritarian leadership is more likely to occur, whereas existing research on quiet quitting has been conducted primarily in Western settings (Harris, 2025). However, studies of this topic in China are limited. Therefore, exploring how employees in Chinese SMEs respond to authoritarian leadership, and how such leadership prompts quiet quitting, is of considerable theoretical and practical value.

To address these gaps, the present study proposes a moderated mediation model in which authoritarian leadership indirectly affects quiet quitting through job burnout, with involuntary presenteeism as the moderating variable. By integrating leadership research, this study provides a nuanced understanding of how authoritarian leadership shapes employees’ psychological states and behavioral outcomes in Chinese SMEs.

## 2 Theoretical background

### 2.1 Authoritarian leadership and job burnout

Conservation of resources theory posits that individuals strive to acquire, maintain, and protect valued resources and that stress emerges when these resources are threatened, lost, or insufficiently replenished (Hobfoll, 1989). Within this framework, authoritarian leadership is a contextual condition that systematically accelerates employee resource depletion. Authoritarianism reflects a leadership style in which supervisors assert absolute authority and demand unquestioned obedience (Cheng et al., 2004). Such leaders often overlook employees’ needs and perspectives, limiting their autonomy and narrowing their opportunities for meaningful participation. These behaviors increase the likelihood that employees will experience unmet expectations and diminished trust in organizational intentions, which can foster cynicism, dehumanization, and psychological states closely associated with emotional exhaustion (Jiang et al., 2019). Chiang et al. (2021) observe that authoritarian leaders often implement coercive tactics such as reprimanding or punishing visible emotional expressions to control their subordinates. This practice fosters the shared belief among team members that emotions are inappropriate, thereby establishing a stable team-level climate for emotional suppression. Forced to suppress their genuine feelings, employees engage in substantial emotional labor that depletes their emotional resources. Drawing on Hobfoll’s (1989) conservation of resources theory, the ongoing drainage of these critical resources generates significant stress and leads to emotional exhaustion, thereby fueling overall burnout. Moreover, authoritarian leaders frequently impose heightened performance pressure and strict behavioral standards, compelling employees to expend substantial cognitive and emotional resources to avoid criticism or punishment. Employees tend to attribute the aggressive behaviors of authoritarian leaders to external factors, such as the leader’s temperament or emotional state, and view themselves as passive recipients of such treatment. This attribution pattern protects them from self-blame or feelings of shame, but simultaneously produces greater psychological strain and diminishes their sense of self-efficacy. These reactions reduce employees’ perceived personal accomplishments and increase their overall level of burnout (Wang et al., 2022). Within the Chinese cultural context, where hierarchical norms are more widely accepted, leadership practices that emphasize control and subordination diminish employees’ self-esteem and confidence, subjecting them to prolonged emotional strain (Zheng et al., 2025). When these intensified efforts fail to yield adequate psychological or material returns, a progressive cycle of resource loss may occur, ultimately leading to increased job burnout (Zhang et al., 2022).

Therefore, we propose the following hypothesis:

*Hypothesis 1: Authoritarian leadership has a positive influence on job burnout.*

### 2.2 Job burnout and quiet quitting

Job burnout is characterized by extreme physical and mental exhaustion, low enthusiasm for work, and emotional fatigue resulting from job responsibilities (Alzoubi et al., 2024). As Hobfoll et al. (2018) state, job burnout results from a gradual loss of valued resources, often manifesting as declining job performance and negative attitudes toward work tasks, rather than being triggered by a single event. Building on conservation of resources theory, when individuals experience burnout, they enter a defensive mode aimed at preserving their remaining emotional and cognitive resources (Hobfoll, 1989; Hobfoll et al., 2018). Conciseness, they are less likely to engage in extra-role behaviors and are more inclined to limit their contributions to minimum requirements of the job (Maslach et al., 2001). Job burnout is conceptualized as a psychosocial phenomenon arising from prolonged exposure to chronic workplace stressors, exacerbated by ineffective coping strategies and insufficient recovery from work demands (Archer et al., 2024). Employees experiencing burnout must divert their resources to manage these pressures, leaving scant resources to enhance their well-being. Consequently, they often disinvest in activities that sustain their well-being, making them more susceptible to decline (Peng and Huang, 2024). This resource depletion and well-being deficit ultimately predispose employees to engage in quiet quitting. High levels of burnout in the workplace are widely regarded as key drivers of the growing prevalence of employees’ quiet quitting (Kang et al., 2023). When experiencing high burnout, employees feel overwhelmed by their job demands, compelling them to withdraw via quiet quitting in search of relief from work-related stress and emotional exhaustion (Lu et al., 2023). Recent empirical findings further confirm that chronic stress, work overload, and burnout are considered primary drivers behind the widespread phenomenon of quiet quitting (Gün et al., 2024). Quiet quitting reflects this withdrawal process because it involves a deliberate reduction of effort to the minimum level required to avoid negative consequences, combined with disengagement from extra-role contributions (Hamouche et al., 2023). When burnout undermines employees’ psychological energy and reduces their capacity for sustained engagement, quiet quitting is a functional means to prevent resource depletion. In this sense, burnout may not simply diminish employees’ well-being; it may also reshape their behavioral strategies by fostering a preference for resource-conserving patterns of minimal engagement. Thus, elevated levels of job burnout create fertile conditions for quiet quitting to occur within the workplace. In this way, burnout serves as a psychological mechanism that links adverse work conditions to employees’ withdrawal of effort, not through overt resignation but through silent behavioral disengagement.

Based on the above reasoning, we advance the following hypothesis:

*Hypothesis 2: Job burnout has a positive influence on quiet quitting.*

### 2.3 Authoritarian leadership and quiet quitting

Managerial recognition and limited opportunities for career development are critical factors contributing to the emergence of quiet quitting behaviors (Pevec, 2023). These findings suggest that when employees perceive limited appreciation for their contributions and see few prospects for professional advancement, they are more likely to psychologically disengage from their roles and restrict their efforts to the minimum level required. Quiet quitting is not necessarily an expression of disdain for one’s work or career, nor is it simple idleness. Rather, it points directly to a toxic organizational culture that damages employee morale and well-being (Hamouche et al., 2023). According to Chu (2014), authoritarian leadership comprises four core elements. It emphasizes strict authority and control, restricts information flow, and closely monitors subordinates. This leadership style also involves undervaluing subordinates’ abilities and showing little interest in input or empowerment. Authoritarian leaders focus on maintaining a strong authoritative image and may manage or manipulate information to sustain their status. Finally, they exhibit didactic behaviors, enforce strict performance and behavioral standards, and often rely on criticism or reprimands when their subordinates fail to meet expectations. Drawing on conservation of resources theory (Hobfoll, 1989, 2001), authoritarian leadership can be conceptualized as a persistent source of resource depletion for subordinates. Authoritarian leaders restrict autonomy, impose unilateral control, and frequently neglect employee needs, creating an environment in which subordinates continuously expend additional emotional and cognitive resources to comply with rigid expectations. As these demands accumulate, employees increasingly seek to protect their remaining resources by minimizing discretionary efforts. Such conditions not only diminish motivation but also increase the likelihood that quiet quitting will occur and persist. Exposure to unclear or contradictory role expectations can lead employees to believe that their efforts are ineffective. This belief erodes motivation and prompts a cycle of reduced investment and psychological withdrawal. Thus, employees may respond through quiet quitting, a disengaged state in which they meet only the basic job requirements while detaching themselves from active involvement (Galanis et al., 2024a,b). Quiet quitting describes a situation in which employees remain in their positions yet contribute only to the minimum level of effort required to avoid sanctions while intentionally withholding participation in extra-role behaviors (Gün et al., 2025b). Prior evidence further indicates that limited autonomy, insufficient career opportunities, reduced perceptions of employee value, and declining organizational trust jointly increase the tendency to engage in quiet quitting (Mahand and Caldwell, 2023). Employees who adopt this behavioral pattern avoid proposing new ideas, refrain from overtime, and do not arrive early on their tasks (Galanis et al., 2024a,b). Taken together, these insights suggest that when authoritarian leadership continually erodes employees’ resources, employees are more likely to quit quiet as a resource-conserving coping strategy.

Taken together, we propose the following hypothesis:

*Hypothesis 3: Authoritarian leadership has a positive influence on quiet quitting.*

### 2.4 Mediating effect of job burnout

Authoritarian leadership, characterized by strict behavioral control, restricted autonomy, and coercive top-down communication, heightens employees’ perceptions of threats to core psychological resources, including control, predictability, and a sense of security. Conservation of resources theory proposes that actual resource loss or the anticipation of further loss elicits protective responses aimed at conserving remaining resources. In such leadership environments, the punitive and inconsistent behaviors of authoritarian leaders create persistent signals of instability, thereby increasing the likelihood of employees experiencing job burnout. Job burnout reflects a sustained state of psychological strain involving emotional exhaustion, depersonalization, and reduced professional accomplishment (Maslach et al., 2001), and indicates the ongoing depletion of emotional and cognitive resources. Consistent with the logic of conservation of resources theory (Hobfoll, 1989), employees experiencing burnout become increasingly motivated to curtail additional resource expenditure to prevent further loss. Burnout functions as a central psychological mechanism that explains how authoritarian leadership shapes subsequent patterns of quiet quitting by employees. Depleted employees are more likely to limit their role engagement and refrain from discretionary contributions, and quiet quitting emerges as a strategic resource conservation response that allows them to maintain minimal functioning while protecting their remaining resources. Chu (2014) show that authoritarian leadership prompts employees to suppress negative emotions, which undermines their well-being and increases psychological strain, thereby heightening stress and emotional exhaustion and intensifying job burnout. This further increases the likelihood that employees resort to quiet quitting as a means of distancing themselves from adverse work conditions (Xueyun et al., 2023). Peng and Huang (2024) note that authoritarian leadership triggers negative emotional experiences in subordinates, ultimately impairing their well-being. Given that both burnout and diminished well-being are closely linked to quiet quitting, the detrimental outcomes of such leadership, which include poor work environments and low organizational commitment, can precipitate this disengaged behavior by exacerbating burnout and undermining employee well-being (Lu et al., 2023). Accordingly, job burnout operates as a key mediating process through which authoritarian leadership translates into heightened quiet quitting behavior.

Therefore, we put forward the following hypothesis:

*Hypothesis 4: Job burnout mediates the relationship between authoritarian and quiet quitting.*

### 2.5 Moderating effect of involuntary presenteeism

Drawing on the job demands–resources model, job demands are closely linked to the process of resource depletion (Demerouti et al., 2001; Bakker and Demerouti, 2007), as employees must invest substantial physiological, cognitive, and emotional effort to cope with high-intensity and high-load work environments. Under such conditions, they may even feel compelled to continue working while being unable to meet these demands, further accelerating the loss of personal resources. Although burnout increases the likelihood of quiet quitting, the magnitude of this effect depends on the conditions that determine employees’ ability to prevent further loss of resources. Involuntary presenteeism refers to situations in which employees feel obligated to attend work despite illness, fatigue, or psychological strain (Johns, 2010). When employees feel forced to work because they fear missing out, falling behind, or facing penalties for their absence, their core psychological needs are unlikely to be met (Van Waeyenberg, 2024). This condition reduces opportunities for recovery and forces employees to continue expending resources that are already in deficit. Consequently, the imbalance caused by burnout becomes more severe in involuntary presenteeism. Employees who cannot step away from work to recuperate may become more inclined to minimize discretionary efforts, which increases the likelihood of adopting quiet quitting as an immediate coping response. Thus, involuntary presenteeism intensifies the resource conservation motive, which links burnout to quiet quitting.

Lu and Cooper (2022) show that employees may feel compelled to work while sick due to external pressures such as stringent attendance policies, increased performance expectations, and apprehension about being judged negatively. When this behavior is driven more by coercion or fear rather than personal commitment, and when intrinsic motivation is lacking, the adverse effects of presenteeism tend to be significantly amplified. Building on this evidence, involuntary presenteeism could be an important contextual moderator shaping the extent to which job burnout translates into quiet quitting. When involuntary presenteeism is high, employees are compelled to attend work despite illness or resource depletion, which exacerbates emotional exhaustion and reinforces their perceptions of continuous resource loss. Under these conditions, the effect of job burnout on quiet quitting becomes stronger because employees have fewer remaining psychological and physical resources, and therefore adopt quiet quitting as a resource-conserving response with greater intensity. By contrast, when involuntary presenteeism is low, employees are less constrained by external pressures to attend work while ill, experience fewer additional demands during periods of burnout, and retain greater discretion in regulating their resource expenditure. As a result, the link between job burnout and quiet quitting weakens because employees face fewer coercive pressures that would otherwise amplify the depletion process.

Based on the above reasoning, we propose the following hypothesis:

*Hypothesis 5: Involuntary presenteeism moderates the positive effect of job burnout on quiet quitting.*

## 3 Methods

Data analysis followed several steps. First, confirmatory factor analysis was conducted using AMOS 26.0 to assess construct validity and discriminant validity among the study variables. Second, reliability analyses, descriptive statistics, and correlation analyses were performed using SPSS 26.0. Third, PROCESS `Macro (Model 4) developed by tested the mediating role of job burnout in the relationship between authoritarian leadership and quiet quitting. Finally, PROCESS Macro (Model 7) examined the moderating role of involuntary presenteeism and the moderated mediating effect. This study used 5,000 bootstrap samples to estimate indirect effects and corresponding 95% confidence intervals.

### 3.1 Sample characteristics

This study develops a moderated mediation model in which authoritarian leadership is specified as the independent variable, job burnout as the mediating mechanism, involuntary presenteeism as the moderating condition, and quiet quitting as the outcome variable. Data were collected through an online survey administered via Wenjuanxing, targeting employees working in Chinese small and medium-sized enterprises (SMEs). Given that SMEs constitute the vast majority of firms and account for a substantial proportion of employment in China, they provide an appropriate and meaningful context for examining the proposed relationships. SMEs may be more susceptible to authoritarian leadership due to their structural and resource characteristics. Compared to large corporations, SMEs often operate with limited formalization, fewer hierarchical layers, and greater centralization of decision-making authority (O'Regan et al., 2005). As a result, leaders tend to rely more on directive and control-oriented behaviors to ensure efficiency and coordination, particularly under resource constraints and environmental uncertainty (Feng et al., 2024). This context makes SMEs a relevant and meaningful setting for examining the effects of authoritarian leadership. To enhance the diversity and robustness of the sample, respondents were drawn from SMEs across multiple industries and organizational contexts. Although precise population-level statistics on the total number of SME employees are not readily available, this multi-industry sampling approach helps improve the heterogeneity and representativeness of the data. Consistent with prior research in organizational behavior, the primary objective of this study is to test theoretically derived relationships among variables rather than to estimate population parameters. Data collection was conducted over a 10-day period from March 26 to April 4, 2025. A total of 363 valid responses were obtained and used for subsequent analyses.

For the data analysis, we first used SPSS 26.0 to examine demographic characteristics, exploratory factor analysis, reliability analysis, descriptive statistics, correlation analysis, and moderation hypothesis analysis. Second, AMOS 23.0, was used to conduct confirmatory factor analysis, discriminate validity analysis, and path analysis. Finally, the SPSS PROCESS Macro 3.4.1 was utilized to test the moderated mediation hypotheses. Regarding the participants’ demographic characteristics, 172 (47.4%) were male and 191 (52.6%) females. Regarding age, 2 (0.6%) participants were aged under 20 years, 148 (40.8%) were aged 20–30 years, 84 (23.1%) were aged 31–40 years, 68 (18.7%) were aged 41–50 years, 57 (15.7%) were 51–60 years, and 4 (1.1%) were over 60 years old. Regarding education, 118 (32.5%) participants held an associate’s degree or lower, 149 (41.0%) held a bachelor’s degree,72 (19.8%) held a master’s degree, and 24 (6.6%) held a doctoral degree. In terms of years of service, 60 (16.5%) participants had worked for less than 1 year, 68 (18.7%) for 1 year to less than 3 years, 33 (9.1%) for 3 years to less than 5 years, 34 (9.4%) for 5 years to less than 7 years, and 168 (46.3%) for 7 years or more.

### 3.2 Measurements

All the measurement instruments employed in this study are well-established international scales with extensive validation in authoritative management and psychology research. These instruments are widely adopted in previous empirical studies, and their reliability and validity have been consistently demonstrated. Given that this study was conducted in a Chinese cultural context, a translation–back translation procedure was adopted to ensure measurement equivalence (Brislin, 1970). The original English scales were first translated into Chinese with careful attention to conceptual and linguistic accuracy. Two researchers with experience in publishing in internationally recognized journals and in-scale translations reviewed the translations and provided guidance to refine and finalize the Chinese versions of the instruments.

The internal consistency of the four key variables was examined, and the results satisfactory reliability for all scales. Specifically, the Cronbach’s alpha coefficient was 0.928 for authoritarian leadership, 0.966 for job burnout, 0.9239 for quiet quitting, and 0.943 for involuntary presenteeism. Cronbach’s alpha values were calculated to assess internal consistency, with coefficients exceeding 0.70 regarded as indicative of adequate reliability (Nunnally and Bernstein, 1994).

Authoritarian leadership is a leadership style in which leaders assert absolute authority and control over subordinates and expect complete and unquestioned obedience (Cheng et al., 2004). This study measured authoritarian leadership using the scale developed by Zhang S. et al. (2024). The scale comprises five items, including statements such as “My leader asks me to obey his or her instructions completely.”

Job burnout is conceptualized as a state in which employees experience severe physical and psychological exhaustion, diminished enthusiasm for work, and emotional fatigue arising from their daily job demands (Alzoubi et al., 2024). In this study, job burnout was assessed using the scale developed by Schaufeli et al. (2020). The instrument consists of eight items, including a representative statement such as: “When I get up in the morning, I lack the energy to start a new day at work.”

Quiet quitting refers to a workplace phenomenon in which employees do not actually resign from their jobs but instead limit their efforts strictly to what is formally required, without engaging in any discretionary or extra-role behaviors (Scheyett, 2023). This construct was measured using the scale developed by Galanis et al. (2023). The scale includes items such as “I do the basic or minimum amount of work without going above and beyond” and “If a colleague can do some of my work, then I let him or her do it.”

Involuntary presenteeism refers to employees’ intention to attend work while ill as a result of external pressures or demands (Van Waeyenberg, 2024). This study employed the measurement scale developed by Van Waeyenberg (2024), which includes items such as “When I do not feel well, I force myself to go to work because I am supposed to.”

**Figure 1:**
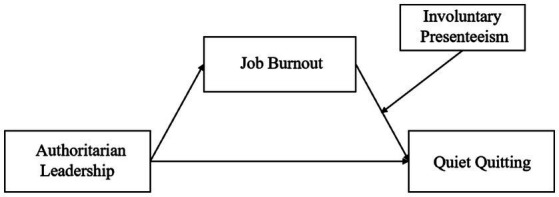
Research model.

All constructs were assessed using a seven-point Likert-type scale ranging from 1 (strong disagreement) to 7 (strong agreement). Higher scores indicate a stronger level of the corresponding psychological tendency. Figure 1 illustrates the overall research framework.

The Figure 1 is the research model.

## 4 Results

### 4.1 Confirmatory factor analysis (CFA)

A CFA was conducted to evaluate the adequacy of the measurement model and compare alternative model structures (Presson et al., 1997). The fit indices were as follows. For the absolute fit indices, the results yielded *χ*^2^ = 437.275 (*p* = 0.000), *χ*^2^/df = 1.988, and RMSEA = 0.052, all of which falls within the acceptable range. In terms of the incremental fit indices, the model showed good fit, with GFI = 0.904, NFI = 0.949, RFI = 0.941, IFI = 0.974, TLI = 0.970, and CFI = 0.974. Collectively, these values indicate that the model achieved an acceptable level of fit.

To further assess the convergent validity, this study examined the average variance extracted (AVE) and composite reliability (CR) of each construct. The AVE values were 0.707 for authoritarian leadership, 0.776 for job burnout, 0.716 for quiet quitting, and 0.808 for involuntary presenteeism, all of which exceeded the recommended minimum of 0.50. Similarly, the CR values were 0.783, 0.901, 0.841, and 0.833, surpassing the threshold of 0.70. According to Jin and Hahm (2021), convergent validity is established when AVE exceeds 0.50 and CR exceeds 0.70. Based on these criteria, all constructs in this study exhibited satisfactory validity. The detailed CFA outcomes are presented in Table 1.

**Table 1:** Confirmatory factor and reliability analyses.

Variable		Estimate	S.E.	C.R.	*p*	Standardized regression weights	Average variance extracted (AVE)	Composite reliability (CR)
Authoritarian leadership (A)	A1	1				0.766	0.707	0.783
	A2	1.021	0.052	19.666	***	0.776		
	A3	1.216	0.066	18.309	***	0.890		
	A4	1.179	0.064	18.421	***	0.895		
	A5	1.207	0.068	17.805	***	0.869		
Job burnout (B)	B1	1				0.857	0.776	0.901
	B2	0.941	0.046	20.343	***	0.824		
	B3	1.051	0.045	23.359	***	0.889		
	B4	1.061	0.045	23.765	***	0.896		
	B5	1.126	0.049	23.092	***	0.883		
	B6	1.093	0.046	23.707	***	0.895		
	B7	1.128	0.047	23.944	***	0.899		
	B8	1.089	0.045	24.084	***	0.901		
Quiet quitting (C)	C1	1				0.847	0.716	0.841
	C2	0.779	0.044	17.700	***	0.711		
	C3	1.028	0.046	22.540	***	0.892		
	C4	1.087	0.047	23.046	***	0.902		
	C5	0.918	0.047	19.435	***	0.818		
	C6	1.099	0.049	22.499	***	0.891		
Involuntary presenteeism (D)	D1	1				0.917	0.808	0.833
	D2	0.942	0.033	28.447	***	0.910		
	D3	0.937	0.037	25.122	***	0.867		
	D4	0.974	0.035	27.644	***	0.900		

Model fit index *χ*^2^(p) = 437.275 (0.000), *χ*^2^/df = 1.988, GFI = 0.904, NFI = 0.949, RFI = 0.941, IFI = 0.974, TLI = 0.970, CFI = 0.974, RMSEA = 0.052. ***p<0.001.

### 4.2 Descriptive statistics and correlation analysis

Descriptive statistics were computed for all study variables, including mean and standard deviation. The average scores for authoritarian leadership, job burnout, quiet quitting, and involuntary presenteeism were 4.511, 4.577, 4.565, and 4.342, respectively. The corresponding standard deviations were 1.618, 1.571, 1.492, and 1.698, respectively.

Correlation analysis indicated that authoritarian leadership was positively associated with job burnout (*r* = 0.369, *p* < 0.001), quiet quitting (*r* = 0.338, *p* < 0.001), and involuntary presenteeism (*r* = 0.348, *p* < 0.001). Job burnout also exhibited a significant positive correlation with quiet quitting (*r* = 0.423, *p* < 0.001) and involuntary presenteeism (*r* = 0.434, *p* < 0.001). In addition, quiet quitting was positively correlated with involuntary presenteeism (*r* = 0.428, *p* < 0.001). Detailed descriptive statistics and correlation coefficients are presented in Table 2.

**Table 2:** Descriptive statistics and correlation analysis.

Variable	Mean	SD	Authoritarian Leadership	Job Burnout	Quiet Quitting	Involuntary Presenteeism
Authoritarian leadership	4.511	1.618	0.707			
Job burnout	4.577	1.571	0.369^***^ (0.136)	0.776		
Quiet quitting	4.565	1.492	0.338^***^	0.423^***^ (0.179)	0.716	
Involuntary presenteeism	4.342	1.698	0.348^***^	0.434^***^	0.428^***^ (0.183)	0.808

***p < 0.001.

To evaluate discriminant validity, we compare the maximum squared correlations among the constructs with their corresponding AVE values based on the conventional criterion that a construct’s AVE should exceed its highest shared variance with the other variables. As Table 2 shows, the parentheses following each correlation coefficient display the square roots of the maximum squared correlations, enabling a direct comparison with the inter-construct correlations. The AVE values were also included to support a straightforward assessment of discriminant validity. Overall, the empirical results were consistent with theoretical expectations: the correlations among the variables were significant in the predicted directions, and discriminant validity was satisfactory.

Potential multicollinearity was examined using linear regression diagnostics in SPSS. The variance inflation factors for authoritarian leadership (1.219), job burnout (1.320), and involuntary presenteeism (1.297) fell well below the commonly accepted cutoff value of 5. These results suggest that multicollinearity did not pose a problem in the analysis.

### 4.3 Hypotheses tests

SPSS PROCESS Model 4 was used to test the mediating role of job burnout. The analysis indicated that authoritarian leadership significantly predicted job burnout (estimate = 0.358, *p* < 0.001) as well as quiet quitting (estimate = 0.194, *p* < 0.001). Job burnout also had a significant positive effect on quiet quitting (estimate = 0.328, *p* < 0.001). Accordingly, Hypotheses 1, 2, and 3 were supported.

Hypothesis 4, which posits that job burnout mediates the relationship between authoritarian leadership and quiet quitting, is also confirmed. The indirect effect was 0.118 and the bootstrapped 95% confidence interval (Boot LLCI = 0.071; Boot ULCI = 0.173) did not include zero, demonstrating a statistically significant mediation effect. Thus, Hypothesis 4 was supported. The detailed hypothesis testing results are presented in Table 3.

**Table 3:** Pass analysis results.

Path	Estimate	SE	*t*	*p*	LLCI	ULCI
Authoritarian leadership → job burnout	0.358	0.048	7.543	0.000	0.265	0.452
Authoritarian leadership → quiet quitting	0.194	0.046	4.194	0.000	0.103	0.285
Job burnout → quiet quitting	0.328	0.048	6.89	0.000	0.234	0.422

Indirect effect of X on Y	Estimate	SE	LLCI	ULCI
Authoritarian leadership → job burnout → quiet quitting	0.118	0.026	0.071	0.173

### 4.4 Descriptive moderating role of involuntary presenteeism

This study further assessed whether involuntary presenteeism affects the association between job burnout and quiet quitting. The moderating effects were evaluated using SPSS PROCESS Macro 3.4.1 (Model 1) with 95% bootstrap confidence intervals based on 5,000 resamples. Table 4 presents the analytical results for Hypothesis 5. Conditional effects were estimated at −1 SD, mean, and +1 SD of the moderator. Because the bootstrap confidence intervals at all three levels excluded zero, the conditional effects were considered statistically significant.

**Table 4:** Moderation analysis results.

Moderator	Level	Conditional effect	Boot SE	*t*	*p*	LLCI	ULCI
Involuntary presenteeism	−1 SD (−1.698)	0.164	0.064	2.555	0.011	0.038	0.291
	M	0.278	0.048	5.834	0.000	0.184	0.371
	+1 SD (1.698)	0.391	0.064	6.092	0.000	0.265	0.517
Interaction: job burnout × involuntary presenteeism		Estimate	Boot SE	*t*	*p*	LLCI	ULCI
		0.067	0.025	2.628	0.009	0.017	0.117

Hypothesis 5 examined whether involuntary presenteeism amplified the link between job burnout and quiet quitting. The interaction term was 0.067, and the corresponding bootstrap confidence intervals (Boot LLCI = 0.017; Boot ULCI = 0.117) did not include zero, thereby supporting the proposed moderating effect.

## 5 Conclusion

This study elucidates the psychological processes that occur when employees of Chinese SMEs are exposed to authoritarian leadership. Drawing on conservation of resources theory and the job demands–resources model, we explain the antecedents and consequences of employee job burnout and delineate the mechanisms through which these effects occur. In particular, this study clarifies how quiet quitting emerges as a behavioral response and how involuntary presenteeism amplifies the effect of job burnout on quiet quitting.

Using empirical data to validate the proposed model, the findings demonstrate a coherent logic: employees under authoritarian leadership are more likely to experience job burnout, which, in turn, increases their propensity for quiet quitting, and involuntary presenteeism strengthens this pathway. These insights have important implications for the sustainable development of Chinese SMEs. The key findings of this study are summarized below.

This study contributes to existing literature in several ways. First, it extends the theoretical framework of quiet quitting by identifying authoritarian leadership and job burnout as the core antecedents. Second, it highlights the boundary conditions under which involuntary presenteeism exacerbates the effect of job burnout toward quiet quitting. Finally, this study offers practical insights for organizations seeking to foster sustainable employee engagement amid rising psychological detachment.

### 5.1 Theoretical implication

Among various leadership constructs, authoritarian leadership warrants particular attention. This focus is driven by two key considerations. First, authoritarian leadership attracts sustained scholarly attention because it is widely recognized as a representative and damaging leadership style that undermines a broad range of outcomes, including team performance and individual initiative, across multiple organizational levels. Its presence in diverse cultural contexts, such as China, Mexico, France, and Russia, further underscores the importance of international firms in emerging markets developing a deeper understanding of this leadership pattern (Lee et al., 2024). This study advances the literature by illuminating the process through which authoritarian leadership shapes employee withdrawal tendencies through a core psychological mechanism. By identifying job burnout as the mediating process linking authoritarian leadership to quiet quitting, the findings reinforce the central proposition of conservation of resources theory. The persistent depletion of personal resources motivates individuals to protect their resources they still possess by reducing their discretionary efforts.

First, this study reveals that authoritarian leadership serves as a significant antecedent of quiet quitting. Employees exposed to authoritarian leadership experience heightened emotional and cognitive strain, which accelerates job burnout and ultimately manifests as quiet quitting as a self-preserving behavioral response. Supervisors exhibiting authoritarian leadership often adopt overbearing and highly controlling approaches that emphasize strict procedures, rigid rules, and hierarchical structures. Such behaviors signal a lack of trust in subordinates’ capabilities and limited respect for their contributions (Chan et al., 2013). The imbalance between rising demands and limited resources increases the likelihood of job burnout, which subsequently contributes to employees’ psychological disengagement from their roles. This disengagement intensifies negative reactions and fosters tendencies to withdraw, ultimately manifesting as quiet quitting (Wan et al., 2018). Authoritarian leadership evokes negative emotional experiences among subordinates and undermines their well-being, both of which are related to quiet quitting. Negative consequences associated with authoritarian leadership, such as diminished organizational commitment and deteriorating work environments, further increase burnout and reduce employee well-being, ultimately contributing to quiet quitting (Peng and Huang, 2024; Lu et al., 2023).

Second, this research demonstrates the mediating effect of job burnout in that the resource drain initiated by a demanding and controlling leader manifests specifically as chronic burnout, which in turn depletes the motivation and energy required for active engagement, making quiet quitting a likely endpoint. Job burnout adversely affects employees’ physical and psychological health and often triggers a vicious cycle. During this cycle, several factors interact and reinforce each other, ultimately leading to sustained psychological distress and diminished job performance (Galanis et al., 2024a,b). Under authoritarian leadership, employees experience a perceived depletion of their personal resources. According to the principle of resource conservation priority, individuals strive to prevent further resource loss. However, the very nature of authoritarian leadership obstructs the replenishment of these resources, leading to continued exhaustion and ultimately triggering job burnout (Peng and Huang, 2024). For employees who experience burnout in a taxing work environment, quiet quitting is a likely response that represents a specific form of coping with persistent difficulties (Galanis et al., 2024a,b). Therefore, this study not only deepens our understanding of the sequelae of authoritarian leadership, but also critically positions job burnout as a pivotal theoretical construct that bridges the gap between leadership-induced stress and a consequential, passive form of employee withdrawal. This process model solidifies the application of stressor-strain-outcome frameworks and the conservation of resources theory in this domain, illustrating a clear pathway: authoritarian leadership acts as a chronic stressor that erodes emotional and mental resources, leading to burnout, which ultimately results in the behavioral outcome of quiet quitting. This finding elevates the theoretical discourse on quiet quitting from a standalone trend to an integral part of the broader nomological network of workplace well-being and leadership, highlighting burnout as an essential, theory-grounded linchpin that connects leadership style to a specific, contemporary form of disengagement.

Third, this finding extends existing models by demonstrating that involuntary presenteeism functions as a critical boundary condition that strengthens the effect of job burnout on quiet quitting. When employees feel obligated to attend work despite fatigue, illness, or psychological strain, their sense of coercion exacerbates resource loss and accelerates the transition from internal depletion to outward withdrawal. Involuntary presenteeism operates as a strain-intensifying contextual factor that magnifies the behavioral consequences of job burnout. Levels of involuntary presenteeism are closely linked to work conditions that involve elevated stress, substantial workload demands, and a serious or restrictive work climate (Miraglia and Johns, 2016). When personal resources are depleted, employees often prioritize attendance to safeguard their health, and many feel compelled to work while ill because of controlling managerial practices, restrictive absence policies, or concerns about potential job losses (Baker-McClearn et al., 2010). Several forms of work-related pressure are positively associated with emotional exhaustion. Prolonged exposure to intensive situational demands increases employees’ cognitive load and arousal levels, subsequently accelerating the development of emotional exhaustion (LePine et al., 2004). As emotional exhaustion intensifies, employees are more likely to experience job burnout. Existing evidence identifies burnout as a meaningful predictor of quiet quitting. When employees face sustained burnout and simultaneously perceive limited opportunities for career advancement, they tend to disengage from work and express withdrawal tendencies by quietly quitting (Xueyun et al., 2023).

Together, these findings advance our theoretical understanding of resource dynamics in high-control leadership settings. The results illustrate that authoritarian leadership triggers a cumulative loss spiral that is especially pronounced under conditions of coerced attendance. This integrated perspective highlights the need for leadership theories to incorporate attendance pressures more explicitly when explaining how employees shift from internal exhaustion to externally observable disengagement outcomes such as quiet quitting.

### 5.2 Practical implication

The findings of this study offer several actionable insights for organizations, especially those operating in high-pressure or fast-growing environments. The phenomenon of quiet quitting has systemic implications for individuals, organizations, and industries. At the individual level, it diminishes job satisfaction, task performance, and psychological well-being while increasing absenteeism, apathy, and professional alienation. The consequences for organizations include reduced productivity, lower customer satisfaction, and heightened talent turnover. Within service-intensive sectors, quiet quitting can further undermine brand reputation, organizational resilience, and long-term competitiveness (Liu-Lastres et al., 2024). Given these wide-ranging repercussions, research on quiet quitting is not merely academic; it is a vital endeavor to develop proactive strategies to safeguard organizational health and ensure sustainable performance.

First, organizations should mitigate authoritarian leadership tendencies by strengthening leadership development and feedback systems. As authoritarian leadership increases employees’ job burnout and, in turn, the likelihood of quiet quitting, firms should invest in training programs that promote supportive, autonomy-enhancing, and participative leadership behaviors. Practices such as 360-degree feedback, leadership coaching, and structured performance conversations can help managers identify controlling tendencies and replace them with more constructive influence strategies. These interventions are particularly important in SMEs, where leaders often rely on directive or compliance-based approaches due to resource constraints. At the same time, organizations should foster a culture of psychological safety and open communication to address burnout proactively (Li et al., 2012). When employees feel safe to express concerns or difficulties, early signals of strain can be identified and managed before escal ating into withdrawal behaviors. To this end, firms may implement regular one-on-one check-ins, anonymous reporting mechanisms, and team-based reflection sessions. Such practices not only help prevent quiet quitting but also enhance long-term organizational resilience by promoting transparency and shared responsibility for employee well-being.

Second, organizations must actively monitor and manage job burnout before it evolves into disengagement or quiet quitting. Establishing early detection mechanisms such as periodic well-being assessments, pulse surveys, or analytics tracking workload patterns can help identify employees at risk of burnout. Managers should be encouraged to redistribute tasks, adjust role expectations, or provide job resources such as autonomy, skill variety, and social support when signs of exhaustion appear. Providing access to counseling services, stress-management workshops, or employee assistance programs can further reduce psychological strain and interrupt burnout withdrawal progression.

Third, firms should carefully review attendance norms and eliminate forms of involuntary presenteeism that exacerbate the impact of burnout on quiet quitting. The results suggest that employees who feel compelled to attend work despite physical or psychological strain are particularly vulnerable to disengagement. Organizations should, therefore, adopt flexible attendance policies, allow sick leave without punitive consequences, and create a culture in which recovery and self-care are legitimized rather than discouraged. Clear communication from leaders that rest is acceptable combined with training for supervisors in managing attendance pressure can reduce involuntary presenteeism and weaken its amplifying effect on withdrawal behaviors.

Fourth, firms should design job structures to reduce chronic strain and promote sustainable engagement. Work redesign initiatives, such as clarifying role expectations, reducing unnecessary bureaucratic demands, implementing fair workload distribution, and offering opportunities for skill development, can directly mitigate the resource depletion triggered by authoritarian leadership. Providing employees with greater decision latitude and encouraging collaborative problem solving can also buffer the negative psychological effects of high-control leadership environments.

Together, these practical measures can help organizations mitigate quiet quitting and its consequences for performance by simultaneously addressing leadership styles, resource depletion, attendance norms, and work design. A holistic approach that targets both the causes and amplifiers of burnout would be more effective than isolated interventions, ultimately supporting a healthier, more motivated, and sustainable workforce.

### 5.3 Limitations and future research

Although this study offers meaningful insights, it has several limitations. These limitations highlight the promising avenues for future research.

First, it employed a cross-sectional design, which limited its ability to make strong causal inferences. Although the theoretical model specifies the progression from authoritarian leadership to job burnout and quiet quitting, temporal ordering cannot be established definitively. Future studies could use multi-wave longitudinal designs or experimental approaches to capture the dynamic nature of the resource depletion and withdrawal processes more accurately.

Second, the data were collected through self-reported measures, which introduce the possibility of common method variance. Although procedural remedies were applied, self-reported data may inflate the relationships between the variables, particularly for constructs related to emotional strain and withdrawal intentions. Future research could incorporate supervisor ratings, peer assessments, objective attendance data, or digital behavioral indicators to strengthen the validity of the findings.

Third, the sample consists primarily of employees from Chinese SMEs, which may limit generalizability. The unique characteristics of Chinese SMEs, such as their collectivist cultural orientation (Zhang L. et al., 2024; Zhang S. et al., 2024) and high power distance (Rui et al., 2024), might shape how authoritarian leadership is perceived and quiet quitting is enacted. For instance, collectivism might suppress overt dissent, channeling disengagement into quieter forms, whereas high power distance could normalize top-down control, altering the baseline against which leadership is judged. Therefore, it is imperative to include more culturally diverse samples in future research. A cross-cultural comparative approach would not only test the generalizability of our model, but also help consolidate an integrated theory that distinguishes universal psychological mechanisms from culturally specific manifestations.

Fourth, this study focused on job burnout as the sole mediating mechanism linking authoritarian leadership to quiet quitting. Although burnout is a theoretically grounded mediator, other psychological processes such as perceived unfairness, relational distrust, or threats to autonomy may also contribute to withdrawal behaviors. Future research could explore multiple parallel mediators or test sequential mechanisms to provide a more comprehensive explanation of how employees translate leadership pressure into disengagement. Future research could examine whether thriving at work acts as a protective factor that mitigates employees’ quiet quitting behavior. Drawing on the conservation of resources perspective, thriving may provide individuals with psychological resources that buffer against withdrawal tendencies under stressful work conditions (Islam and Idris, 2025).

Fifth, this study examines involuntary presenteeism as a single boundary condition; however, other contextual factors may further influence the burnout–quiet quitting relationship. In addition, prior studies have highlighted that the relationship between leadership and employees’ behavior may vary depending on contextual or interpersonal factors (Rubel et al., 2025). Workplace norms regarding flexibility, organizational justice, team climate, or individual differences such as resilience or coping styles may either intensify or buffer the effects identified in this study. Future research could adopt a multilevel approach to investigate how broader organizational or cultural systems interact with individual experiences of resource depletion.

## Data Availability

The datasets presented in this article are not readily available because the datasets generated and/or analyzed during the current study are available from the corresponding author upon reasonable request. Requests to access the datasets should be directed to XJ, soohua1005@gachon.ac.kr.
